# Evaluation of artificial intelligence for pulmonary embolism detection on CTPA: A single-center retrospective study comparing AI, radiologists, and AI-assisted interpretation

**DOI:** 10.1097/MD.0000000000050543

**Published:** 2026-09-04

**Authors:** Huiyang Zhang, Minjie Dong, Hongbo Li, Xiang Li, Yan Jiang, Yulei Wan

**Affiliations:** a Department of Radiology, The Sixth Hospital of Wuhan, Affiliated Hospital of Jianghan University, Wuhan, Hubei, P.R. China; b Department of Radiology, The Central Hospital of Wuhan, Tongji Medical College, Huazhong University of Science and Technology, Wuhan, Hubei, P.R. China.

**Keywords:** artificial intelligence, computed tomography pulmonary angiography, pulmonary embolism

## Abstract

Pulmonary embolism (PE) is a life-threatening condition commonly diagnosed with computed tomography pulmonary angiography (CTPA). Although artificial intelligence (AI) has been applied for PE detection, its performance relative to physician-only and AI-assisted interpretation remains incompletely characterized. The study aimed to evaluate the diagnostic performance and reading time across 3 CTPA interpretation strategies: AI alone, physician-only reading, and AI-assisted physician reading. We retrospectively analyzed CTPA images from 50 patients diagnosed with PE at a single center and randomly divided them into 2 groups: one group (n = 25) was reviewed by radiologists from different levels, and the other group (n = 25) was reviewed by radiologists with AI assistance. Then, AI independently reviewed all images. The reference standard was established by consensus among 3 senior radiologists. Diagnostic performance and reading time were compared across the 3 reading paradigms. AI alone achieved an overall sensitivity of 91.24% and precision of 96.12%; sensitivity declined from 100% in grade 1 to 2 vessels to 86.32% in grade ≥6 vessels. Compared with physician-only reading, AI-assisted reading increased sensitivity and precision for junior radiologists (64.28%–75.51% and 81.82%–89.16%, respectively) and intermediate radiologists (80.67%–92.34% and 88.47%–97.31%, respectively). AI assistance also reduced mean reading time at both experience levels (both *P* < .05). In this small, single-center retrospective exploratory study, AI-assisted CTPA interpretation was associated with higher lesion-level sensitivity and precision and shorter reading times than physician-only interpretation. Larger prospective multicenter studies are required to validate these findings and assess their generalizability.

## 1. Introduction

Pulmonary embolism (PE) is a common acute cardiovascular condition, with an annual incidence of approximately 39 to 115 cases per 100,000 people.^[1,2]^ Without timely diagnosis and treatment, mortality may reach 20% to 30%, making early detection essential.^[3]^ Although catheter pulmonary angiography has historically served as a reference standard, its invasive nature and risk of complications limit routine use.^[4–6]^ Computed tomography pulmonary angiography (CTPA) depicts the location, extent, and morphology of pulmonary emboli and has become the first-line imaging modality for patients with suspected PE. CTPA images can be converted to 3-dimensional (3D) images by multiple planar reconstruction (MPR) to provide a more comprehensive and 3D view, which is valuable for confirming or excluding PE.^[7]^.

Advances in artificial intelligence (AI), deep learning, and convolutional neural networks have enabled automated analysis of thoracic computed tomography (CT) images.^[8–11]^ For PE assessment, AI systems may help identify and localize filling defects throughout the pulmonary arterial tree. This task is time-consuming for radiologists and may be affected by subtle distal emboli, overlapping arteries and veins, contrast-flow artifacts, and respiratory motion.^[12]^ Previous studies have reported promising performance of AI-assisted CTPA interpretation, but evidence regarding its added value across readers with different experience levels remains limited.^[13]^

This study compared 3 reading strategies for PE detection on CTPA – AI alone, radiologists alone, and radiologists with AI assistance – to determine whether AI support improves diagnostic accuracy and efficiency. We also analyzed the causes of false-positive and false-negative AI findings.

## 2. Materials and methods

### 2.1. General information

This single-center, retrospective exploratory study included 50 consecutive patients diagnosed with PE at our hospital between October and December 2025. It was designed to preliminarily assess the potential value of PE-AI software in assisting radiologists with CTPA interpretation in a real-world clinical setting, focusing on diagnostic performance and reading efficiency, rather than establishing broad clinical generalizability.

We randomly divided the enrolled patients into group A (radiologists group, 25) and group B (radiologists combined AI group, 25) according to the random number table method after anonymous processing. This study was reviewed and approved by the Ethics Committee of Wuhan Central Hospital (NO. 2020 03). The requirement for informed consent was waived because of the retrospective study design.

The inclusion criteria were as follows: patients diagnosed with PE in our hospital; complete imaging data in the picture archiving and communication system; high image clarity and ability to meet diagnostic requirements. The exclusion criteria were as follows: incomplete imaging data saved in AI software; AI software was unable to effectively calculate and analyze the images of patients with PE; severe motion artifacts and poor quality of images leading to difficulty in diagnosis; after discussion by 3 associate chief physicians or higher-level radiologists, a definitive diagnosis could not be made for the case.

All enrolled patients were imaged at our hospital with a Philips 256-slice spiral CT machine (Philips) and an Ante dual-tube high-pressure injector (Shenzhen Antech Industry Co., Ltd.). The contrast agent used was iodoethylene alcohol (370 mg/mL), injected at a flow rate of 4.0 to 4.5 mL per second to a total of 60 to 70 mL, followed by 30mL normal saline water injected at the same flow rate using a Surestart intelligent trigger (Philips, Best, the Netherlands; trigger threshold was 90–100 Hu). Scan range: from lung apex to lung base, acquiring CTPA images of the pulmonary arterial phase. Scan parameters: detector configuration of 0.5 × 128 layers, tube voltage of 120 kV, tube current of 300 to 350 mAs, scan time of 6.5 to 8.0 seconds, field of view of 250 mm × 250 mm, acquisition matrix of 512 × 512, and slice thickness of 0.5 to 1.0 mm.

### 2.2. Image analysis

Three image interpretation strategies were used. Resident radiologists engaged in image diagnosis served as the junior physicians group, and attending radiologists who engaged in diagnosing chest CT for 5 years served as an intermediate physicians group. They independently diagnosed the CTPA images of 25 patients in group A using a double-blind method. Resident radiologists and attending physicians in group A were assisted by the PE-AI software to diagnose the CTPA images of 25 patients in group B using a double-blind method. All CTPA images were reviewed by radiologists independently, without access to patient clinical information, to ensure an unbiased assessment of both AI-assisted and manual interpretations. All CTPA datasets from the 50 enrolled patients were additionally analyzed by the AI software alone. The AI software used in this study was the United Imaging Intelligence AI software (United Imaging, version 2025 R001). The software was used to automatically detect suspicious thrombi on CTPA images, outline suspected emboli on the images using color-coded markings, and provide structured output including thrombus location, thrombus volume, and vessel occlusion status. As a commercial product, detailed information on model architecture, training datasets, and external validation procedures is proprietary and not fully available to the authors. The collected results detected by the AI were divided into 6 groups according to the grade of vessel branch in which the pulmonary thrombus was located. We designated grade 1 as the main pulmonary artery trunk; grade 2 as the right and left pulmonary arterial trunks; grade 3 as the lobar arteries; grade 4 as the segmental arteries; grade 5 as the subsegmental arteries of the lung segments; and grade 6 and above as the lower level of the subsegmental arteries of the lung segments, as well as the more finely branched arteries.

The original CTPA images were analyzed by 3 associate senior and higher-level imaging specialists. The CTPA images of PE features show a filling defect in the lumen of the pulmonary artery vessel, which can be classified as central, eccentric, appendage, and complete according to their presentation.^[14,15]^ Their reading results were used as the “gold standard,” and in case of disagreement, 3 physicians voted to reach a final conclusion.

### 2.3. Statistical analysis

The observation indicators we use are sensitivity, precision, detective accuracy (compliance rate), false positive, false negative, and reading time. Sensitivity tests whether the method can accurately identify the presence of pulmonary thrombus. Precision, equivalent to positive predictive value, was calculated as TP/(TP + FP) and represented the proportion of positive detections that were true-positive lesions.^[16]^

Statistical analyses were performed using SPSS version 27.0 (IBM Corp., Armonk). The normality of continuous variables was assessed using the Shapiro–Wilk test before analysis. Count data were expressed as n (%), and the mean ± standard deviation was used for the measurement data that conformed to normal distribution, and the median and interquartile spacing M (P25, P75) were used for those that did not; continuous variables conformed to normal distribution using the *t* test, and those that did not conform to normal distribution using the nonparametric test, and categorical variables using the χ^2^ test, and *P* < .05 was considered a statistically significant difference.

## 3. Results

The baseline characteristics of the enrolled patients are summarized in Table 1. Group A and group B each included 25 patients. The median age was 72 years in group A and 71 years in group B, and the proportions of male patients were 60% and 64%, respectively. There were no significant differences in age or sex distribution between the 2 groups (*P* > .05).

**Table 1:** Baseline characteristics of the enrolled patients.

Variable	Overall cases	Group A	Group B	Statistic (*P* value)
Figure	n = 50	n = 25	n = 25	
Age, yr, median (P25, P75)	72 (63, 78)	72 (64.5, 75)	71 (59.5, 79)	50 (*Z* value = 0.34, *P* = .734)
Range	32–90	39–90	32–87	–
Sex, n (%)				
Male	31 (62)	15 (60)	16 (64)	50 (χ² value = 0.085, *P* = .771)
Female	19 (38)	10 (40)	9 (36)	

The reference standard established by consensus among 3 senior radiologists, a total of 434 pulmonary thrombi were identified in 50 patients with confirmed PE: 2 in grade 1 vessels, 21 in grade 2, 61 in grade 3, 140 in grade 4, 115 in grade 5, and 95 in grade ≥6 vessels.

AI detected 412 thrombi, of which 396 were true positives. Overall sensitivity was 91.24%, precision was 96.12%, and the compliance rate was 88.00% (Table 2). Sensitivity was 100% in grade 1 and grade 2 vessels and decreased to 98.36%, 90.71%, 90.43%, and 86.32% in grades 3, 4, 5, and ≥6, respectively. Precision by vascular level is reported in Table 2.

**Table 2:** Diagnostic performance of AI alone for pulmonary artery thrombus detection by vascular level.

Vascular classification	Reference standard, n	Number of AI detection	Number of AI correctly detect (TP)	Number of FP	Number of FN	Sensitivity, % TP/(TP + FN) [95% CI]	Precision, % TP/(TP + FP) [95% CI]	FP, % FP/(TP + FP) [95% CI]	FN, % FN/(TP + FN) [95% CI]	Compliance rate, % TP/TP + FN + FP [95% CI]
Level 1	2	2	2	0	0	100% [34.24–100.00]	100% [34.24–100.00]	0	0	100% [34.24–100.00]
Level 2	21	21	21	0	0	100% [84.54–100.00]	100% [84.54–100.00]	0	0	100% [84.54–100.00]
Level 3	61	62	60	2	1	98.36% [91.28–99.71]	96.77% [88.98–99.11]	3.23%	1.64%	95.24% [86.91–98.37]
Level 4	140	131	127	4	13	90.71% [84.76–94.49]	96.95% [92.41–98.81]	3.05%	9.29%	88.19% [81.90–92.49]
Level 5	115	111	104	7	11	90.43% [83.68–94.57]	93.69% [87.55–96.91]	6.31%	9.57%	85.25% [77.89–90.46]
Level 6 and above	95	85	82	3	13	86.32% [77.98–91.83]	96.47% [90.13–98.79]	3.53%	13.68%	83.67% [75.11–89.69]
Total	434	412	396	16	38	91.24% [88.21–93.55]	96.12% [93.79–97.60]	3.88%	8.76%	88.00% [84.67–90.69]

Sensitivity (%) = TP/(TP + FN) × 100%.

Precision/PPV (%) = TP/(TP + FP) × 100%.

FP (%) = FP/(TP + FP) × 100%.

FN (%) = FN/(TP + FN) × 100%.

Compliance rate (%) = TP/(TP + FN + FP) × 100%.

AI = artificial intelligence, CI = confidence interval, FN = false negative, FP = false positive, PPV = positive predictive value, TP = true positive.

Diagnostic performance across the 3 interpretation strategies is summarized in Table 3. AI alone achieved 91.24% sensitivity and 96.12% precision. Without AI, junior radiologists achieved 64.28% sensitivity and 81.82% precision, whereas intermediate radiologists achieved 80.67% sensitivity and 88.47% precision. With AI assistance, sensitivity and precision were 75.51% and 89.16% for junior radiologists and 92.34% and 97.31% for intermediate radiologists, respectively.

**Table 3:** Comparison of diagnostic performance among AI alone, physician-only reading, and AI-assisted physician reading.

	AI	Junior physicians	Junior physicians + AI	Intermediate physicians	Intermediate physicians + AI
Sensitivity, % (95% CI)	91.24% (88.21–93.55)	64.28% (58.02–70.11)	75.51% (69.04–81.00)	80.67% (75.18–85.18)	92.34% (87.76–95.31)
Precision, % (95% CI)	96.12% (93.79–97.60)	81.82% (75.67–86.69)	89.16% (83.52–93.03)	88.47% (83.55–92.07)	97.31% (93.86–98.55)
FN rate, %	8.76%	35.72%	24.49%	19.33%	7.66%
FP rate, %	3.88%	18.18%	10.84%	11.53%	2.69%

AI = artificial intelligence, CI = confidence interval, FN = false negative, FP = false positive.

Reading times are shown in Table 4. For junior radiologists, mean reading time decreased from 620.04 ± 96.76 seconds without AI to 543.60 ± 63.22 seconds with AI (*P* = .002). For intermediate radiologists, mean reading time decreased from 518.00 ± 65.76 seconds to 367.36 ± 49.98 seconds (*P* < .05).

**Table 4:** Comparison of reading time (seconds) between physician-only and AI-assisted physician reading.

Comparison	Physician-only reading, time (s) (X̅ ± SD)	Physician + AI reading, time (s) (X̅ ± SD)	*t* value	*P* value
Junior physicians	(620.04 ± 96.76)	(543.60 ± 63.22)	3.307	.002
Intermediate physicians	(518.00 ± 65.76)	(367.36 ± 49.98)	9.120	<.001

AI = artificial intelligence, FN = false negative, FP = false positive, X̅ ± SD = mean ± standard deviation.

**Figure 1.**
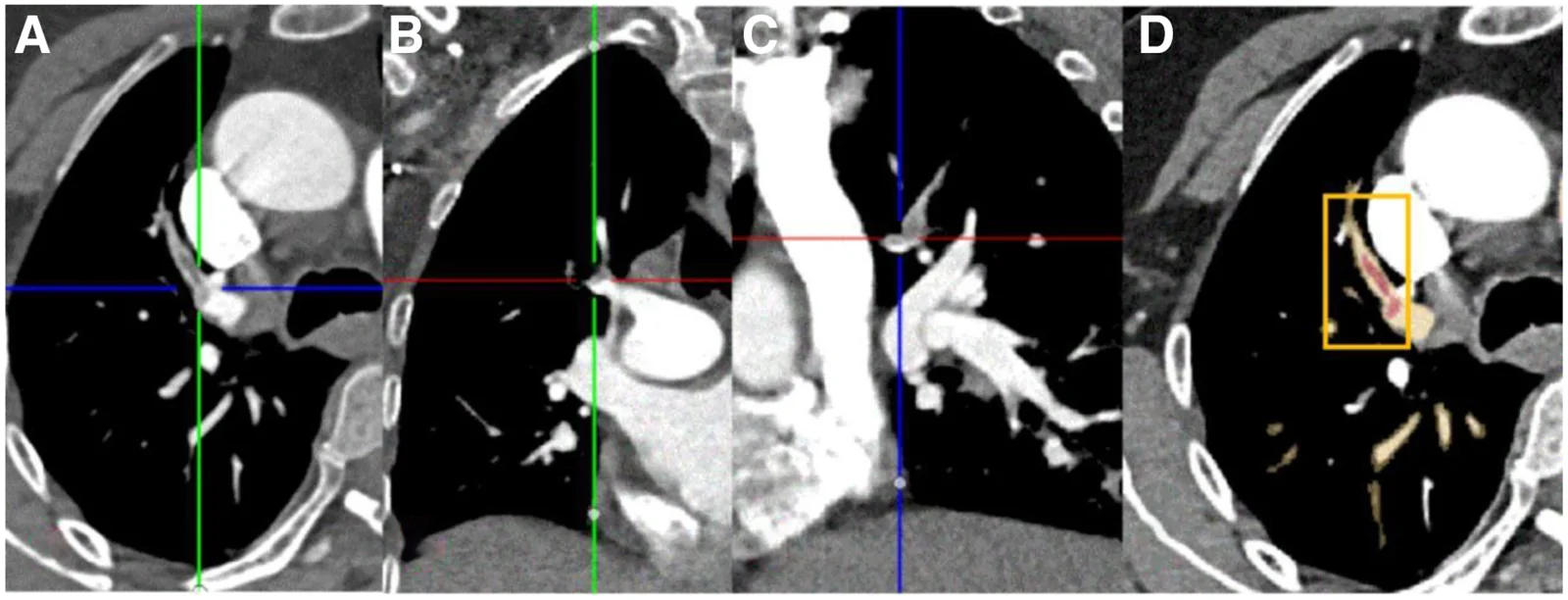
True-positive AI detection. Panels (A–C) show the thrombus on axial, coronal, and sagittal CTPA images, respectively; panel (D) shows accurate detection by the AI system. AI = artificial intelligence, CTPA, computed tomography pulmonary angiography.

**Figure 2.**
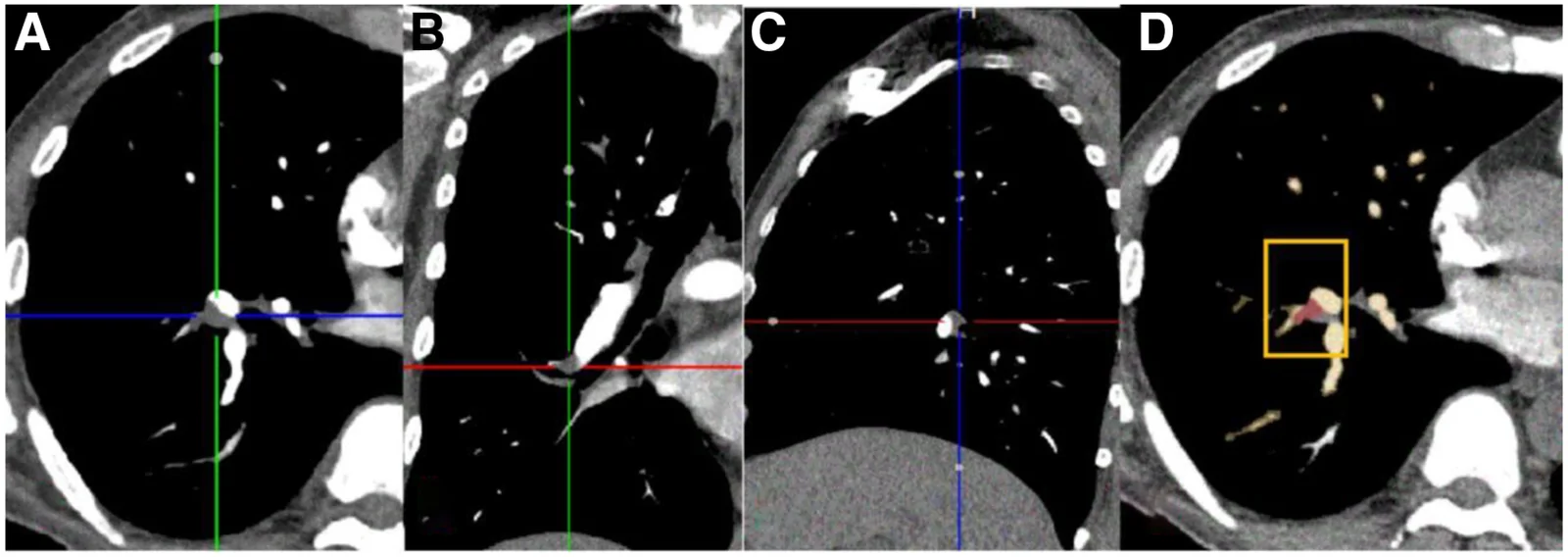
False-positive AI detection caused by an adjacent pulmonary vein. Panels (A–C) show the pulmonary vein without an embolus; panel (D) shows the region incorrectly identified as a thrombus by the AI system. AI = artificial intelligence.

**Figure 3.**
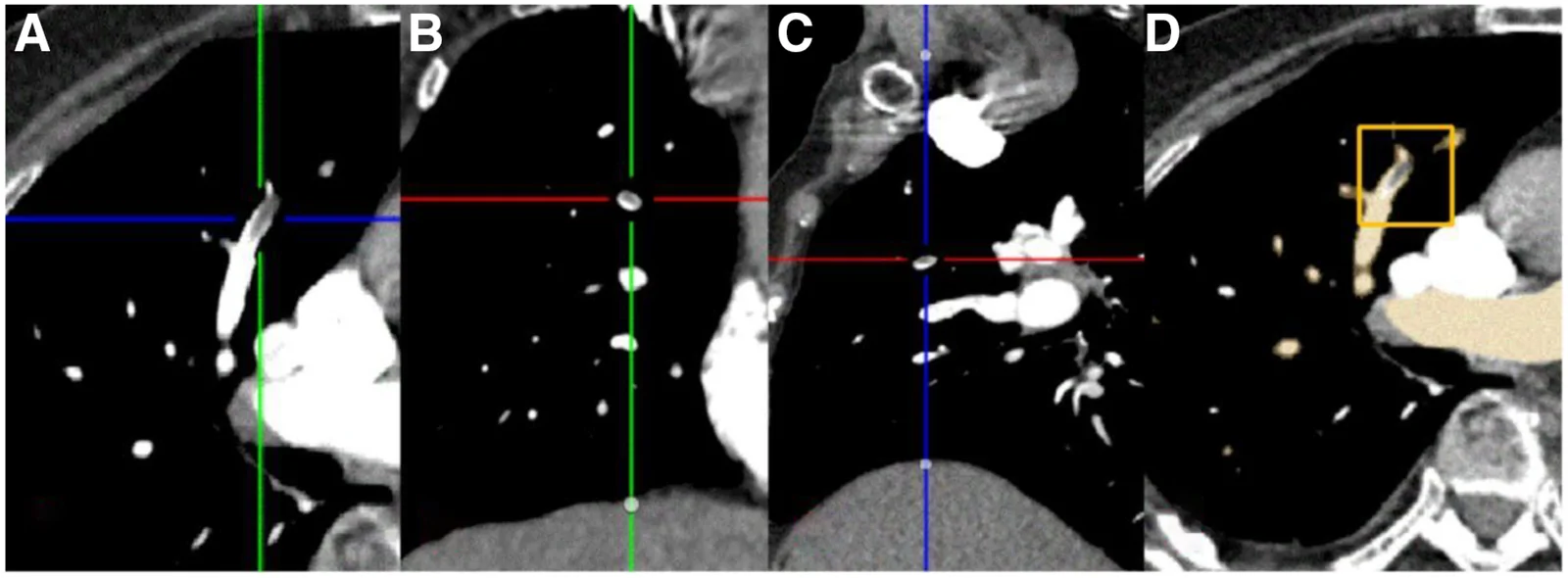
False-negative AI finding in a distal pulmonary artery. Panels (A–C) show the thrombus; panel (D) shows that the lesion was not identified by the AI system. AI = artificial intelligence.

The PE-AI detection software marked the size of the thrombus and rendered the area where they existed. In all figures, panels “A to C” were axial, coronal, and sagittal images after 3D reconstruction of the original images, with the locus line marking the focal area. The results of the AI software for the same patient and the same part of the axial images were represented by panel “D.” Representative imaging examples are shown in the figures. These figures illustrate true-positive (Fig. 1), false-positive (Fig. 2), and false-negative (Fig. 3) AI findings and demonstrate how the system identifies thrombi on CTPA.

## 4. Discussion

AI has increasingly been applied to vascular imaging in recent years, including deep-learning-based pulmonary vessel segmentation and artery–vein separation.^[17–22]^ In this exploratory study, AI alone showed high lesion-level sensitivity and precision for PE detection, and AI-assisted interpretation was associated with higher diagnostic performance and shorter reading times than physician-only interpretation. The highest observed performance occurred when intermediate radiologists used AI support. These findings support the potential role of AI as an adjunct to, rather than a replacement for, radiologist judgment.

One important finding was that AI performance varied by vascular level. Sensitivity, precision, and compliance rate were highest in larger vessels and generally declined in smaller, more distal branches, whereas false positives and false negatives increased. This pattern may reflect the reduced vessel caliber and smaller thrombus size in distal pulmonary arteries.

**Figure 4.**
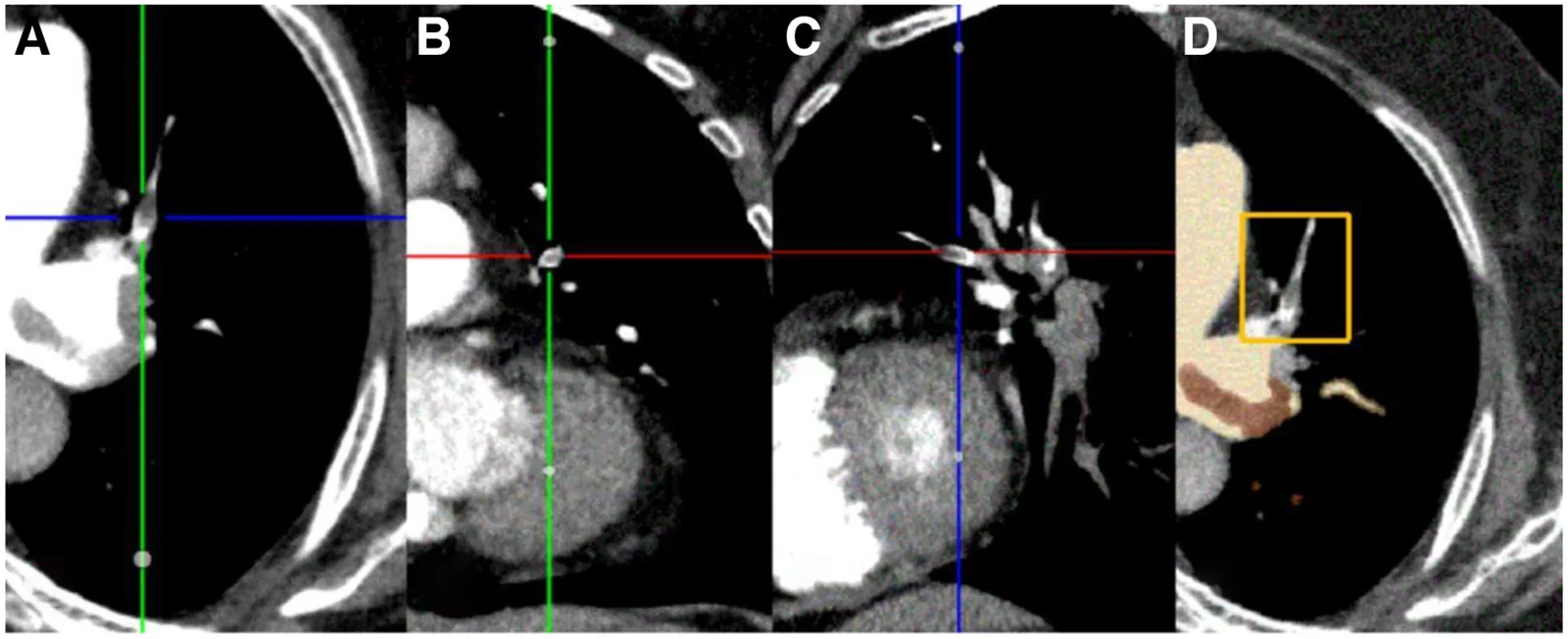
False-negative AI finding associated with complete vascular occlusion. Panels (A–C) show the occlusive thrombus; panel (D) shows that the lesion was not identified by the AI system. AI = artificial intelligence.

**Figure 5.**
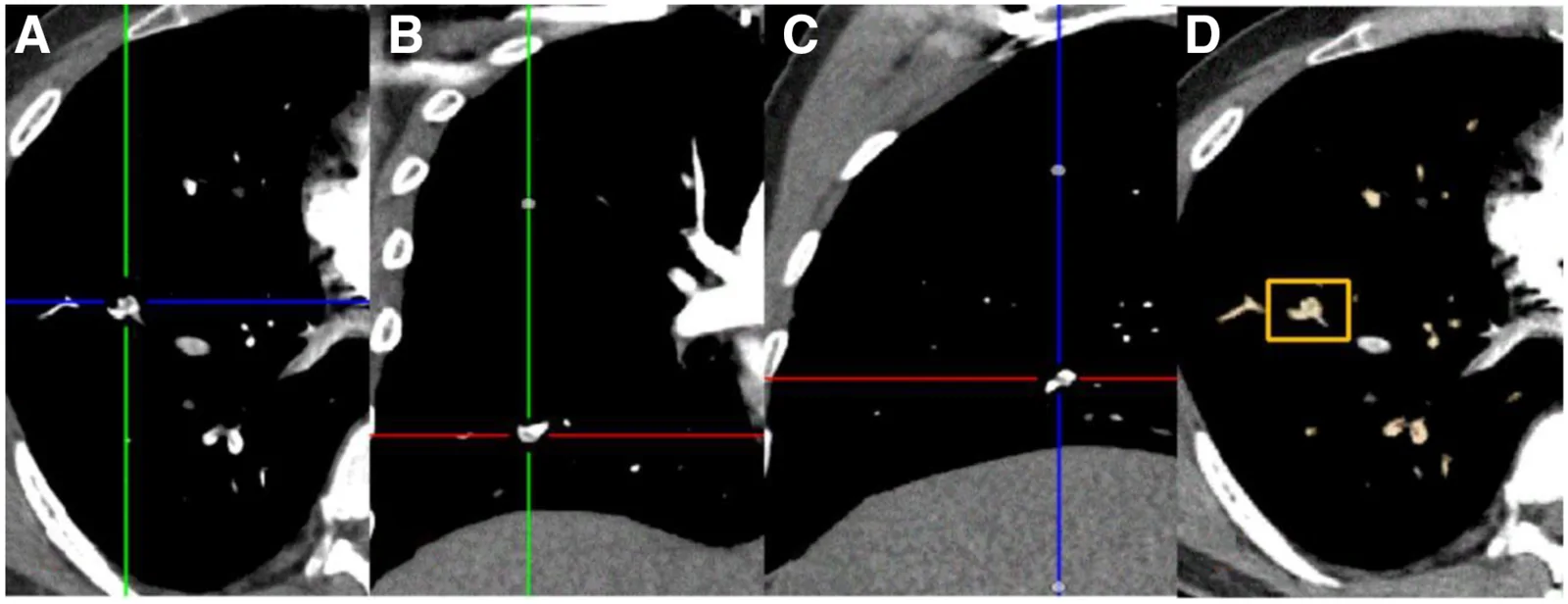
False-negative AI finding at a pulmonary arterial bifurcation. Panels (A–C) show a thrombus crossing the bifurcation; panel (D) shows that the lesion was not identified by the AI system. AI = artificial intelligence.

**Figure 6.**
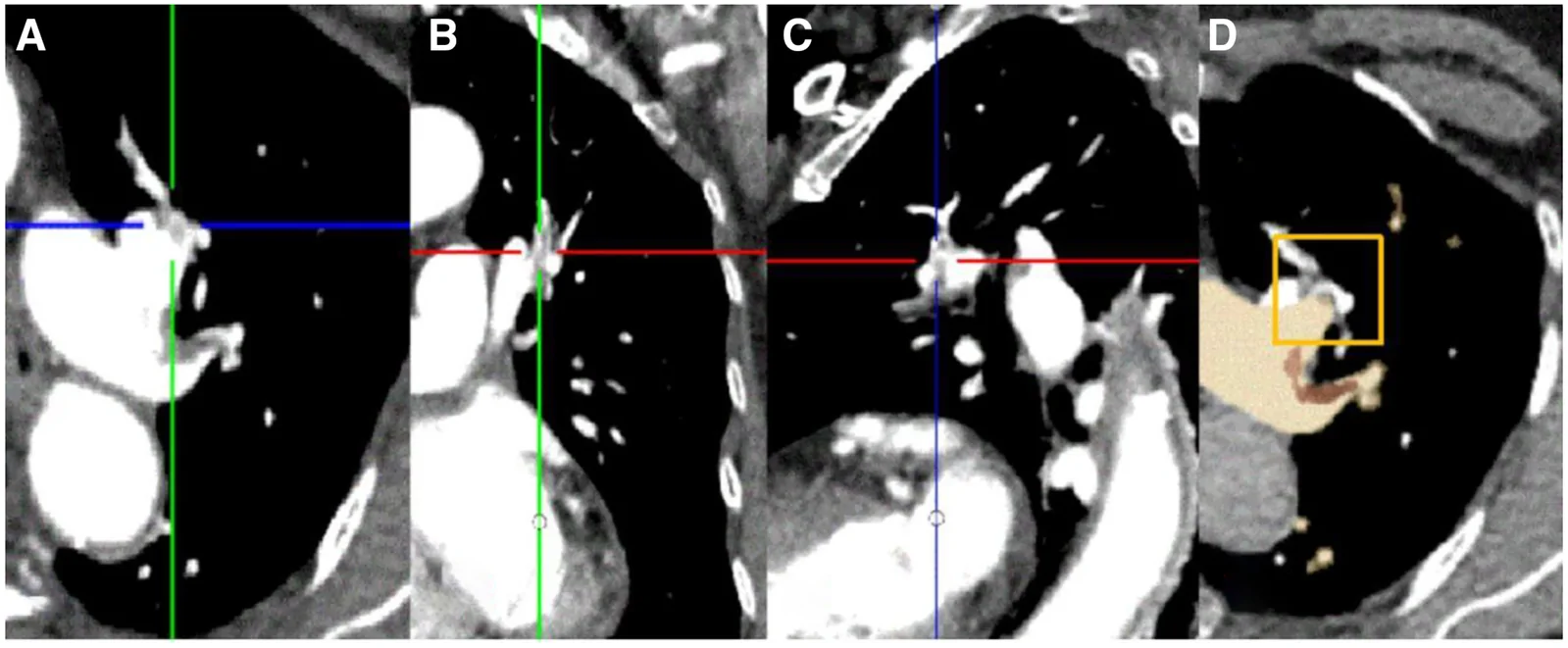
False-negative AI finding involving a subtle thrombus. Panels (A–C) show the lesion; panel (D) shows that the thrombus was not identified by the AI system. AI = artificial intelligence.

**Figure 7.**
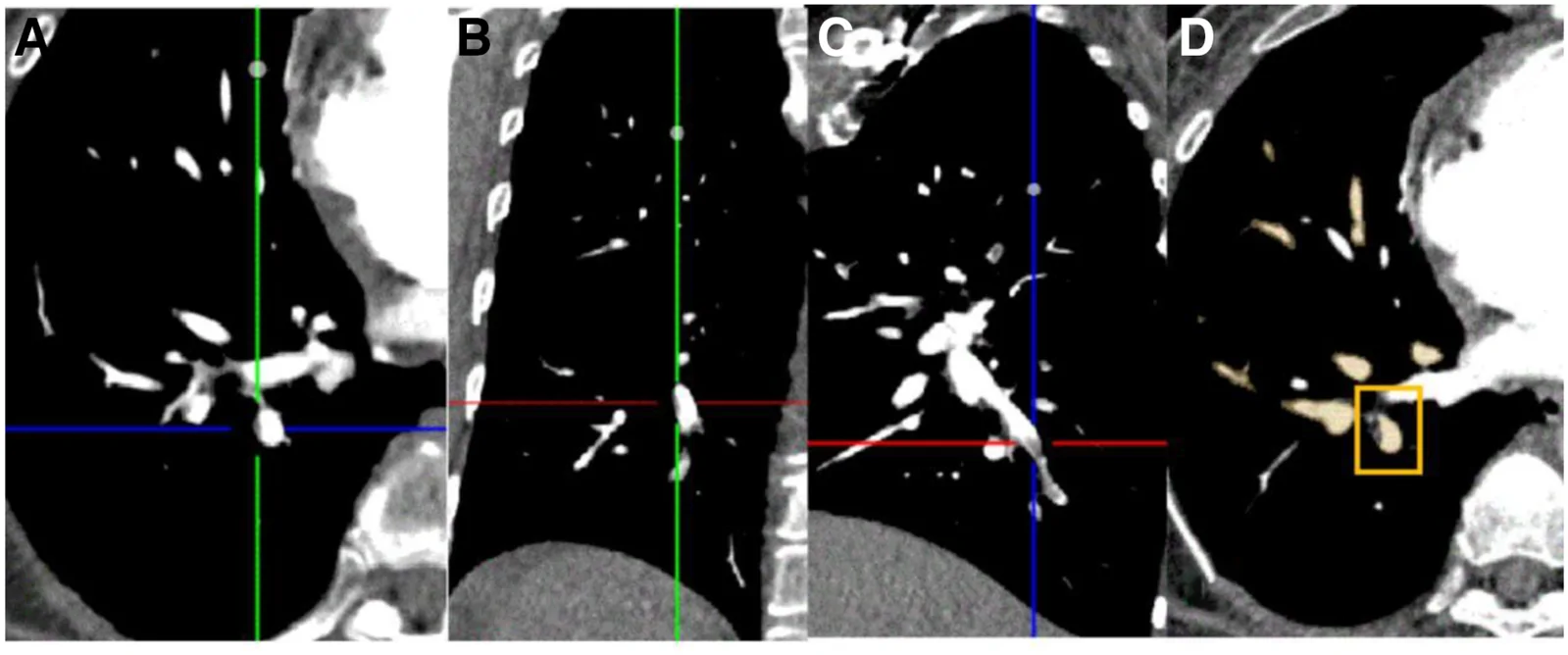
False-negative AI finding involving a thin mural thrombus. Panels (A–C) show the lesion, which is most conspicuous on sagittal MPR; panel (D) shows that the thrombus was not identified by the AI system. AI = artificial intelligence.

**Figure 8.**
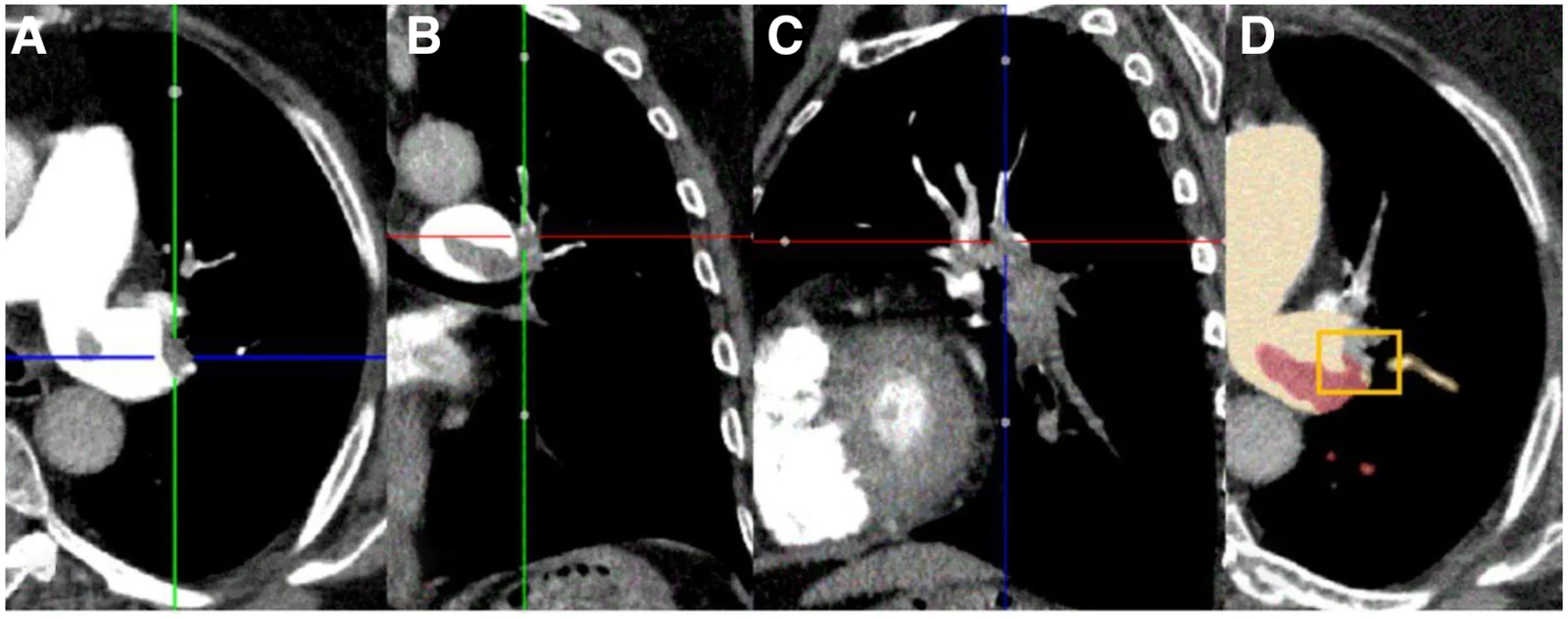
False-positive AI finding caused by a small hilar lymph node adjacent to the pulmonary artery. Panels (A–C) show the lymph node; panel (D) shows the region incorrectly identified as a thrombus by the AI system. AI = artificial intelligence.

**Figure 9.**
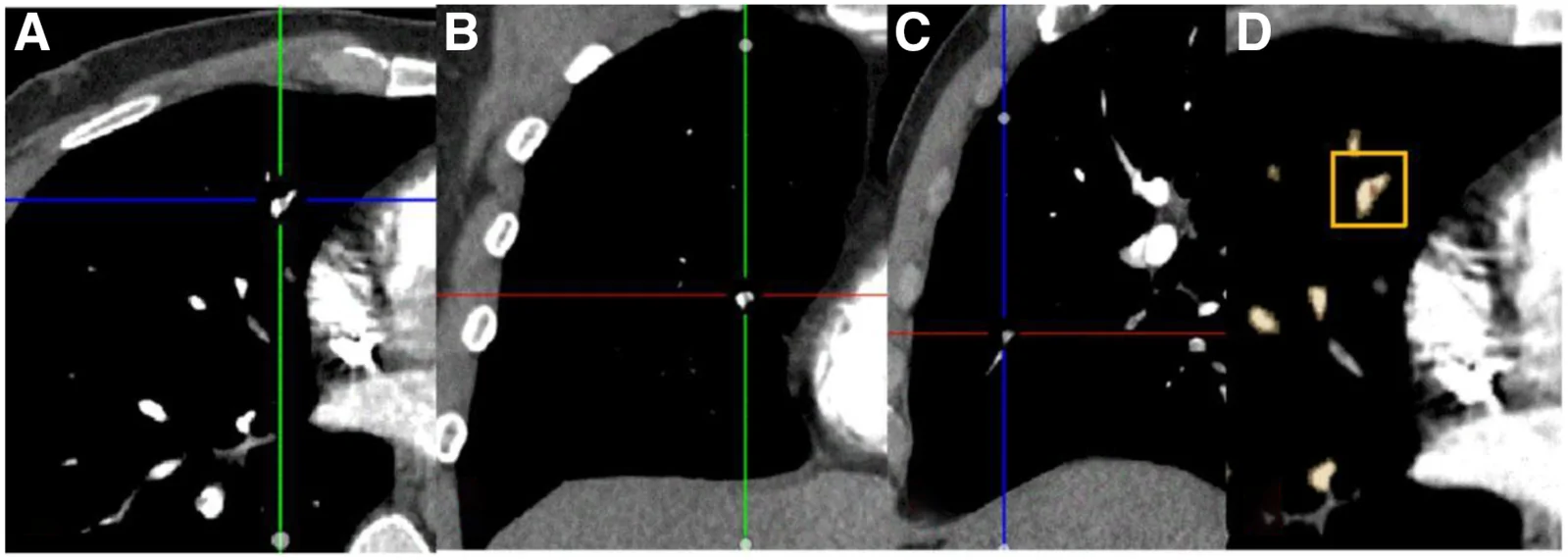
False-positive AI finding at a small distal pulmonary arterial bifurcation. Panels (A–C) show the bifurcation; panel (D) shows the region incorrectly identified as a thrombus by the AI system. AI = artificial intelligence.

**Figure 10.**
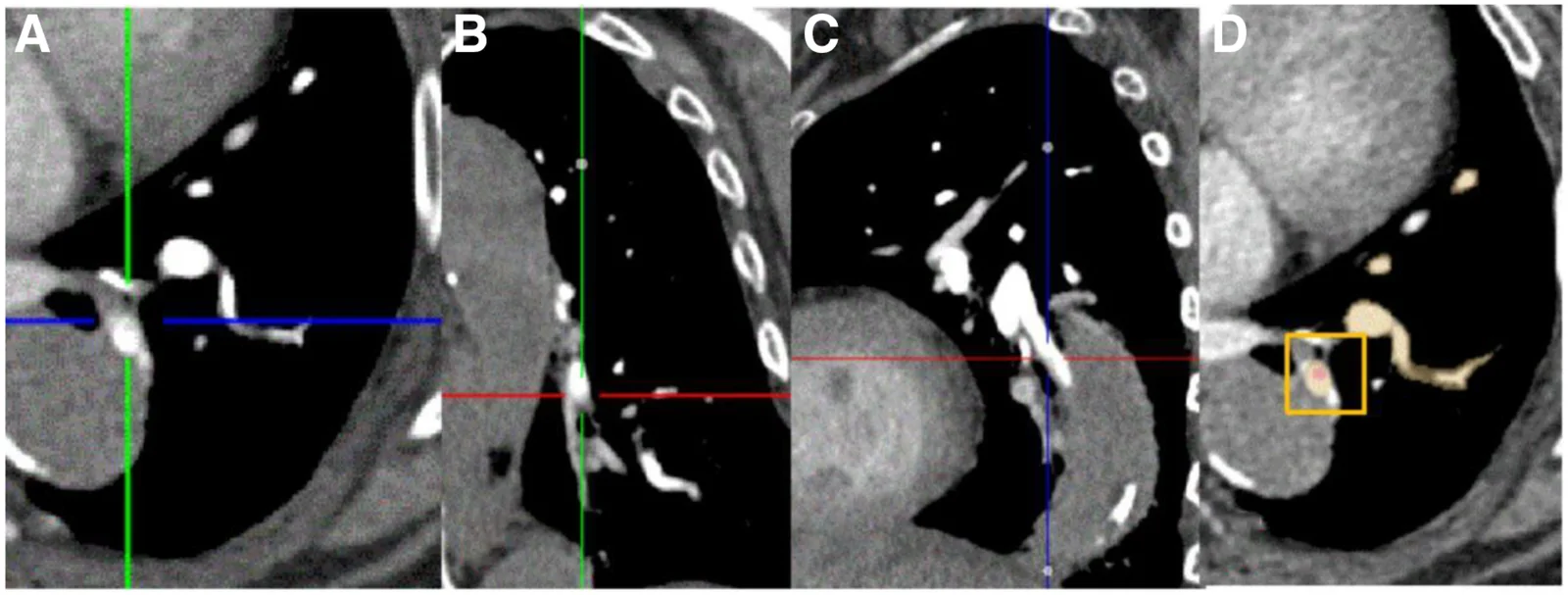
False-positive AI finding related to motion or flow artifact. Panels (A–C) show a small artifactual shadow adjacent to a mildly displaced vessel; panel (D) shows the region incorrectly identified as a thrombus by the AI system. AI = artificial intelligence.

In Table 2, we find the PE-AI detection software performs with 16 false positives and 38 false negatives in 50 samples. We compare and analyze the original images of each missed or misdiagnosed embolus with the results of PE-AI detecting. Among the 38 false-negative lesions, 9 were located in distal small pulmonary arteries that show a partial filling defect, 12 were associated with complete proximal occlusion and absent distal contrast filling, 7 crossed a pulmonary arterial bifurcation, 5 had differences in the composition of the thrombus, and 5 were with a thin attached wall that were more conspicuous on MPR images (Figs. 3–7).^[4,23]^ The 16 false-positive detections were attributed to pulmonary veins adjacent to arteries (3/16), small hilar lymph nodes (1/16), distal arterial bifurcations (9/16), and respiratory or flow-related artifacts (3/16; Figs. 2 and 8–10). Multiplanar assessment may help distinguish these mimics and identify thrombi that are subtle on axial images.^[24]^ PE-AI software relies heavily on axial images for recognition, and some thrombi can show better on MPR; if the AI is able to recognize them in all 3 orientations, it may be possible to improve diagnostic accuracy and compliance even further.

AI assistance improved the observed performance of both junior and intermediate radiologists, with the greatest increase among intermediate readers (Table 3). The software may support interpretation by highlighting suspicious embolic foci and prompting closer inspection of numerous pulmonary arterial branches. The combination of intermediate radiologists with AI was associated with the highest diagnostic performance among all reading strategies, including AI alone. Nevertheless, radiologist review remains essential because AI output requires verification, particularly in examinations affected by artifacts or complex vascular anatomy. In this setting, AI may be most useful as a second reader or decision-support tool.

AI assistance was also associated with shorter mean reading times for both experience levels. The reduction was greatest for intermediate radiologists, who also showed the largest diagnostic performance gain with AI support. Although the system produced false-positive and false-negative findings, its use may help streamline CTPA review; this potential workflow benefit requires confirmation in larger prospective studies.

The AI system was a commercial product, and detailed information regarding its model architecture, training datasets, and external validation procedures was not available to the authors. Consequently, the technical robustness and generalizability of the model could not be independently assessed, and the findings should be interpreted in the context of this limitation.

While the AI system used in this study is a commercial tool, and detailed information on its model architecture, training datasets, and validation is proprietary. Although AI performance in assisting radiologists was demonstrated, the generalizability and technical robustness of the model cannot be fully assessed. Readers should interpret the results in the context of this limitation.

## 5. Limitations and conclusion

This study has several limitations, and further research is needed. The study was a single-center, retrospective analysis with a relatively small sample size (n = 50), which may limit the statistical precision and generalizability of the findings. Conducting a prospective study with a larger sample size would provide more robust evidence. Diagnostic performance may be influenced by reader experience, as the study included both resident and attending radiologists with different levels of expertise. Future studies could standardize training or stratify by experience level. Although the AI software provides standardized outputs, interpretation of false-positive and false-negative results may still be affected by subjective judgment. Future research could incorporate consensus reading or automated adjudication to reduce variability. The AI system used is a commercial product, and detailed information regarding model architecture, training datasets, and external validation is proprietary. These potential biases were considered in the design and interpretation of the study, and readers should interpret the results cautiously.

In conclusion, in this single-center retrospective study, PE-AI software was associated with improved diagnostic performance and shortened reading times compared with physician-only interpretation. These findings suggest that AI may serve as a supportive tool in clinical application. Given the exploratory nature of the study, limited sample size, and single-center design, the results should be interpreted cautiously and should not be overgeneralized. Further validation in larger, prospective, multicenter studies with external datasets is required to confirm the potential clinical utility of AI-assisted CTPA interpretation.

## Author contributions

**Conceptualization:** Huiyang Zhang, Hongbo Li, Xiang Li, Yulei Wan.

**Formal analysis:** Huiyang Zhang, Hongbo Li.

**Investigation:** Huiyang Zhang.

**Methodology:** Huiyang Zhang.

**Data curation:** Minjie Dong, Xiang Li, Yan Jiang.

**Software:** Xiang Li, Yulei Wan.

**Supervision:** Yulei Wan.

**Writing – original draft:** Huiyang Zhang, Yulei Wan.

**Writing – review & editing:** Huiyang Zhang, Yulei Wan.
